# Tannate of Quinia

**Published:** 1847-10

**Authors:** John McLean

**Affiliations:** Prof., &c.; Jackson, Mich.


ILLINOIS AND INDIANA

MEDICAL AND SURGICAL JOURNAL.

Vol. II. NEW SERIES.—OCTOBER, 1847. No. 4.

### PART I.—ORIGINAL COMMUNICATIONS.

### ARTICLE I.

*Tannate of Quinia.* By JOHN McLEAN, M. D., Prof., &c.

Tannic acid has a powerful affinity for quinia, and produces by union with it an insoluble tannate. So readily do they unite, that it becomes one of our most delicate tests for the presence of this alkali. When a solution of the sulphate is so largely diluted that the addition of ammonia will scarcely produce any cloudiness of the solution, tannic acid will cause a copious flocculent precipitate of the tannate of quinia.

Sweden has a law, that all cinchona bark imported into that country shall be tested by an infusion of galls, and if it does not produce a precipitate of tannate of quinia, the bark is rejected as worthless. Here, I would make the remark, that much of the pulverized bark found in the shops of this country, is nearly, and in many cases entirely destitute of quinia, which may readily be determined by this simple test.

The best cinchona barks contain cinchonia quinia and tannic acid, with several other proximate principles; but the acid, owing to some peculiarity, or to its action being modified by some other constituent of the bark, does not chemically unite with the vegetable alkalies. The term tannic acid is not applied to any one proximate principle, invariable in its character and composition; but to a set of substances somewhat different in their nature, but closely allied to each other, and possessing in common many properties. The tannic acid obtained from certain vegetables, when mingled with a solution of a sesquisalt of iron produces a dark brown precipitate and colors the liquid blue; but when obtained from certain other vegetables, it produces a grayish-green precipitate with the same solution, and colors the liquid green. The first is found in galls, and the different species of oak; the second in Peruvian bark, catechu and various other vegetables.

Nut galls contain a large proportion of tannic acid and yield it more readily than any other vegetable matter, hence, it is more frequently obtained from this source than any other, and is frequently distinguished by the name of galls-tannic acid.

*Preparation.*—Tannate of quinia may be prepared, by adding to an infusion of cinchona, an infusion or tincture of galls, so long as any precipitate is produced. When thus prepared it is quite impure, containing coloring and other matters. It may however be obtained pure, directly from the bark by a process more complicated. The readiest, although not the cheapest method of procuring the pure article, is to precipitate it from a clear solution of sulphate of quinia, by adding that of tannic acid, so long as any precipitate is produced, which when dried is fit for use. This is the method I have usually followed in preparing the article for my own practice.

The disulphate of quinia is compounded as follows:

| | Atoms. | Eq. vol. |
|---|---|---|
| Sul. Acid, | 1 | 40 |
| Quinia, | 2 | 324 |
| Water of chrystallization, | 8 | 72 |
| Chrystallized disulphate of quinia, | 1 | 436 |

Now, in order to form the tannate, two atoms of tannic acid are required, to unite with two of quinia, in the one equivalent of the disulphate (the atomic weight of which is 436). The atomic weight of tannic acid being 212, that of two, would be 424, which would give us nearly equal parts of it and the disulphate; but in consequence of the impurity of the tannin of commerce, a much greater proportion of it is required.

Tannate of quinia is composed as follows:

| | Atoms. | Eq. vol. | per cent. |
|---|---|---|---|
| Quinia, | 1 | 162 | 43.31 |
| Tannic Acid, | 1 | 212 | 56.69 |
| Tannate of Quinia, | 1 | 374 | 100.00 |

From the above it will be seen that more than half of it is composed of tannnic acid.

*Properties.*—Insoluble in water; soluble in acetic and muriatic acids; has a yellowish white color and a slightly bitter taste.

*Medical properties and uses.*—Dr. Otto affirms that he has cured obstinate cases of intermittents by the agency of this preparation, which had resisted the use of sulphate of quinia and other powerful remedies. He also states that he found it very useful in typhus, general weakness, and tendency to putrescency, where the sulphate of quinia seemed to be ineffectual. (See *Dunglison's New Remedies*, page 534.)

The experiments of Dr. Nonander, Secretary to the Swedish Medical Association, go to establish the belief that the tannic acid of the cinchonas is necessary to their full febrifuge powers.

Pereira, in his work on Materia Medica, vol. 2, p. 437, says: " The tannate of quinia is declared, by Dr. Nonander, of Stockholm, to be the most powerful of the quinia salts. The tannic acid, though not the peculiar febrifuge constituent of cinchona bark, yet contributes to its tonic powers, and thereby promotes the activity of the alkaloids. This statement is supported by the already referred to remark of Berzelius, (see p. 432,) that the most active cinchonas are those which contain the largest quantity of tannin."

"There exists a law in Sweden," (says Berzelius, Traité de Chim. v. 587,) "in virtue of which every cinchona bark imported into that country is tested by the infusion of galls, and the porsulphate of iron, a solution of gelatine and emetic tartar; and it is proved by an experience of more than sixteen years, that the most efficacious bark is that which precipitates the most strongly a solution of gelatine and emetic tartar; in other words, that which contains the most tannin." (Pereira's *Mat. Med.* vol. 2, p. 432.)

Dr. Hauff has recently administered this preparation in intermittent neuralgias and fevers, and succeeded in cases where the sulphate of quinia had failed. (See *Ranking's Half-Yearly Abstract* for July–Dec. 1845, p. 346.)

In the summer of 1846, I was first induced to use it, in intermittents accompanied with diarrhœa and an irritable condition of the stomach, and the result was so favorable that I not only continued its use in such cases, but extended it to simple intermittents, and that state of general weakness, accompanied with a relaxed surface and profuse perspiration, which frequently is a sequence of this disease. In every case of simple intermittents, where it was administered, the disease was as readily arrested as it would have been by the use of the sulphate. I have used it in many cases of intermittents, and have known of its being thus employed by others, and generally with the same result.

When given for a considerable length of time, it did not produce any constipation. There is but little to be feared from this effect, for the astringent properties of the tannic acid is greatly modified by its chemical union with the quinia. When administered in intermittents, accompanied with diarrhœa and an irritable stomach, we do not give it in preference to the sulphate on account of any astringent effect it may exert upon the bowels, but because it is less apt to irritate the already irritated mucous membranes. If, while using this preparation, it becomes necessary to obtain the astringent effects of tannin upon the bowels, it should be given in extra doses; and if while using the sulphate, (as it will chemically combine with the quinia,) it should be given in such quantities as to be in excess.

It is particularly serviceable in those cases of debility, attended with profuse perspiration, which frequently follows intermittent fevers. I have given it in cases of this kind with great benefit, and think it much better than the other salts of quinia, having arrested the profuse perspiration, and produced a more healthy action of the surface with this than with any other article. Where the use of quinia has been indicated in cases of anemia, accompanied with an irritable state of the stomach and bowels, this preparation has been used with a happy effect. Although having used it to some extent in cases of protracted intermittents, I have not tested it sufficiently, to speak of its powers in such cases, of permanently arresting the disease.

If this is not a more powerful febrifuge than the sulphate, there are several conditions in which it might be used to a greater advantage.

Its nearly tasteless quality, (it possessing but a very slight degree of bitterness,) renders it particularly valuable for the use of children, and such others as possess an irritable stomach, and to whom the sulphate of quinia becomes disgusting on account of its bitter taste.

As this salt is soluble in muriatic and acetic acids, its effects may be quickened by giving a weak solution of either soon after it is taken. This article is easily prepared, and might be afforded at a much cheaper rate than the sulphate.

*Dose.*—The same as of the sulphate of quinia.

Jackson, Mich., Aug. 24, 1847.

